# Association between rheumatoid arthritis and pulpal-periapical pathology: a systematic review

**DOI:** 10.1007/s00784-023-05305-7

**Published:** 2023-10-12

**Authors:** Ertugrul Karataş, Ayhan Kul, Josette Camilleri, Zehra Yonel

**Affiliations:** 1 Department of Endodontics, Faculty of Dentistry, Atatürk University, Erzurum, Turkey; 2 Department of Physical Medicine and Rehabilitation, Faculty of Medicine, Atatürk University, Erzurum, Turkey; 3 School of Dentistry, College of Medical and Dental Sciences, University of Birmingham, Birmingham, UK; 4 Periodontology Research Group, School of Dentistry, College of Medical and Dental Sciences, University of Birmingham, Birmingham, UK

**Keywords:** Rheumatoid arthritis, Apical periodontitis, Systemic diseases, Oral health

## Abstract

**Objectives:**

Rheumatoid arthritis (RA) is a debilitating disease where numerous pro-inflammatory cytokines have a proven role in its pathology. These cytokines are also involved in the pathogenesis of apical periodontitis (AP) where they have a pro-inflammatory role and induce bone resorption. Patients with RA may therefore be more prone to develop pulpal-periapical pathology (PPP). This study systematically reviewed the existing literature evaluating the association between RA and PPP.

**Materials and methods:**

Studies including human participants with both RA and PPP were included. The search was performed in PubMed, Web of Science, and The Cochrane Library databases using keywords and Medical Subject Headings (MeSH) search terms. The risk of bias was assessed using Newcastle–Ottawa Quality Assessment Scale. The following parameters were extracted and analyzed by the reviewers; author, journal, year, design of the study, diagnostic criteria for periapical pathology, the association between rheumatoid arthritis and periapical pathology, and the evidence level.

**Results:**

The search identified 142 records. Inclusion criteria were as follows; studies in the English language, including human participants only, including patients with RA and PPP, cohort studies, cross-sectional studies, clinical trials, and case–control studies. According to the inclusion criteria, 5 studies were included in this systematic review. Three of the five studies reported significant association between RA and PPP.

**Conclusions:**

Existing evidence suggests there may be an association between RA and PPP.

**Clinical relevance:**

Clinicians should be aware that RA patients can be more prone to develop PPP which may result in a reduced quality of life.

## Introduction

According to the World Health Organization, almost half of the world’s population is affected by dental caries and globally in 2010, US$ 442 billion was spent on the management of caries, periodontitis and replacement of teeth lost for these two diseases [1]. Untreated dental caries causes inflammatory changes in the dental pulp which results in pulpal inflammation and infection [2]. The pulpal inflammation/infection can be managed with partial or full pulpotomy [3, 4] or root canal treatment; otherwise, inflammation/infection may extend beyond the apex of the tooth leading to PPP/AP [2].

If AP is not adequately managed, then the continued presence of irritants in the apical part of the root canal system may result in acute inflammation gradually shifting to a chronic inflammatory reaction, known histologically as a periapical granuloma. Clinically, this is usually seen as an asymptomatic radiolucency and it reflects a state of quiescence, or “balance” with the microbes being confined to the root canal [5, 6]. AP may lead to a chronic abscess development with a sinus tract or it may possibly exacerbate into an acute abscess [7]. There is also emerging evidence that AP may lead to increased systemic inflammation (higher plasmatic levels of CRP, IL-6 and fibrinogen) if left untreated [8, 9]. Moreover, several studies suggest a potential association between AP and systemic diseases such as; type 2 diabetes [10, 11], ankylosing spondylitis [12], cardiovascular disease [13], and RA [14].

RA is the most common form of inflammatory joint disease, with a global prevalence of 1%. It is characterized by symmetrical involvement of the peripheral joints and symptoms include progressive joint destruction, swelling stiffness, and pain [15]. Although the exact cause of RA is unknown, recent findings suggest that autoimmunity and genetics would be the reason for the disease development [16–18].

The dominant feature in the RA is inflammation of synovium which occurs as a consequence of leukocyte infiltration into the synovium [19, 20]. As a result of this inflammation, the synovial capillary flow decreases because of the increased fluid volume which results in the hypoxia of the interior inflamed synovium [21] and the synovial membrane becomes hyperplastic [19]. Increased levels of cytokines which plays a central role in the perpetuation of synovial inflammation and the infiltration of inflammatory and immune cells such as: B and T lymphocytes, macrophages, and dendritic cells are present [19, 22]. Numerous studies reported that there is an association between the periodontitis and RA [23–26]. While some authors have explained this association through the higher titres of serum rheumatoid factor [25, 27] or increased cytokines levels that are believed to play a crucial part in causing periodontal breakdown [25], others suggested that the activity of some periodontal pathogens, such as P*orphyromonas gingivalis*, is the cause of the association [28, 29]. According to the authors, *P. gingivalis* is able to induce protein citrullination by releasing a specific deaminase, which might result in the stimulation of anti-citrullinated protein antibodies formation in RA patients [26, 30, 31].

Numerous pro-inflammatory cytokines such as; interleukin (IL)-1, IL-6, IL-12, IL-17, tumour necrosis factor α, RANK, and RANK ligand are associated with elevated serum levels and have a proven role in the pathology of RA [32, 33]. These cytokines are also involved in the pathogenesis of AP, where they play a pro-inflammatory role and induce bone resorption [34–36].

Several studies evaluated the possible association between RA and periapical pathology. Though there is some dissonance within the reported literature with some studies reporting an association between RA and periapical pathology [14, 37], and others refuting this [38]. A systematic review evaluated the possible association between periapical pathology and autoimmune disease, such as type I diabetes mellitus, inflammatory bowel disease, and RA, and reported an association between AP and autoimmune diseases [39]. However, different autoimmune diseases are characterised by different pathogenesis. Moreover, some studies reported no association between RA and diabetes [40, 41]. This is an indication that autoimmune diseases should be evaluated separately.

Currently, there is no systematic review that correlates the possible association between RA and PPP. Therefore, the aim of the present study was to perform a systematic review of the prevalence of PPP in RA patients compared to healthy subjects.

## Research question

Does the presence or absence of RA affect the prevalence of PPP in adult patients?

## Materials and methods

### Protocol guidelines followed

The present systematic review followed the guideline of the Preferred Reporting Items for Systematic Reviews and Meta-Analysis (PRISMA 2020) [42]. The systematic review protocol has also been registered in the prospective register of systematic reviews (PROSPERO) (CRD42022384369).

### Review question and objective

This review aimed to assess the possible associations between RA and PPP. The research question of the present systematic review was formulated based on the Population Intervention Control Outcome (PICO) strategy:

- Participants: Adult patients;
- Interventions: Assessment of periapical health in patients with RA;
- Comparator: Assessment of periapical health in patients without RA;
- Outcome: Prevalence of PPP.

### Outcome measures

The primary outcome measure for the present study was to establish the prevalence of PPP in patients with RA.

### Inclusion and exclusion criteria

Inclusion criteria.

- English language studies
- The period between 1950 – December 2022
- Studies including human participants only
- Studies including the prevalence of PPP both in patients with RA and in healthy control subjects.
- Cohort studies
- Cross-sectional studies
- Clinical trials
- Case–control studies

Exclusion criteria.

- Non-English language studies
- Animal studies

### Search strategy

The following keywords were used in conjunction with the Boolean Operator “OR”: "apical periodontitis" "periapical disease" "periradicular disease" "periapical pathology" "pulpal pathology" "apical abscess" "periapical lesion" "periapical abscess" “root canal treatment” “endodontic*” and were searched for with the key-term "rheumatoid arthritis". (((((((((("apical periodontitis") OR ("periapical disease")) OR ("periradicular disease")) OR ("periapical pathology")) OR ("pulpal pathology")) OR ("apical abscess")) OR ("periapical lesion")) OR ("periapical abscess")) OR ("root canal treatment")) OR ("endodontic*")) AND ("rheumatoid arthritis").

**Fig. 1:**
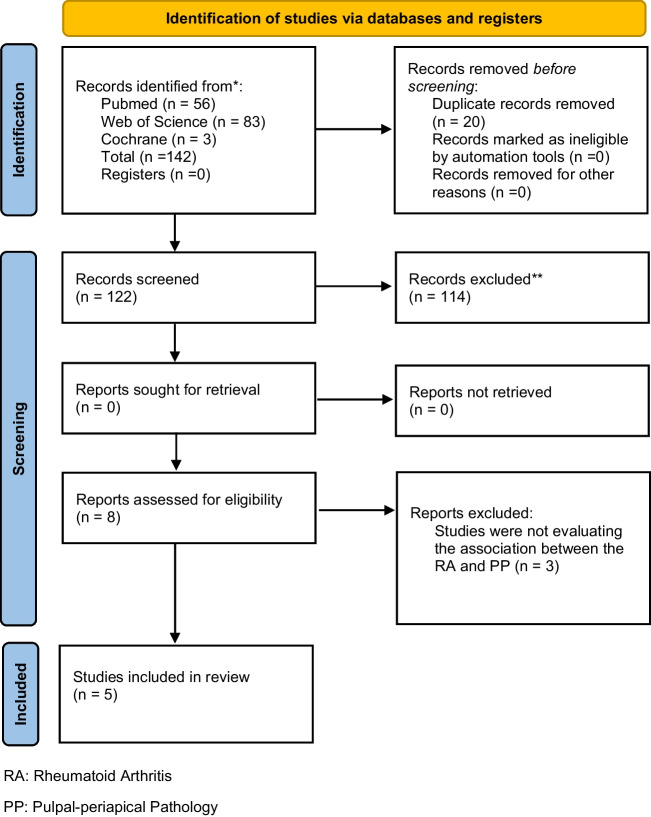
PRISMA diagram of search process

The search was performed in different databases including PubMed, Web of Science, and The Cochrane Library with by 2 different reviewers screening titles and abstracts of the studies. The selected studies were checked for possible duplication and the studies meeting the criteria were evaluated via full text screening. The study search process is shown in Prisma 2020 flow diagram [43] (Fig. 1).

### Data extraction and data management

Prior to completing the data extraction, a pilot data extraction form was developed two reviewers independently extracted data on a random sample of returned articles and a kappa coefficient was determined to ensure inter-relater reliability. Once satisfied with the form and extraction process full data extraction was undertaken. The following information were extracted and analyzed by the reviewers, author, journal, year, design of the study, sample size and groups, diagnostic criteria for periapical pathology, association between RA and periapical pathology, results of interest, and the evidence level.

### Risk of bias

Risk of bias was assessed independently by two reviewers (E.K and A.K) using Newcastle—Ottawa Quality Assessment Scale (an adapted version for cross-sectional studies) [44]. The original Newcastle–Ottawa Scale was also used for the assessment of the cohort and case–control studies. Any disagreements between the two reviewers was resolved by consulting with a third author [ZY].

### Data synthesis

A quantitative synthesis was not possible due to the extensive heterogeneity observed through statistical testing as demonstrated by the Chi-square (46.21) and *I*^2^ (91%) tests across the included studies. Thus, a narrative synthesis was performed to combine the findings of the included studies.

## Results

### Search process

The electronic databases search resulted in a total of 142 records. Of these, 83 were found in Web of Science, 56 in PubMed, and 3 in The Cochrane Library databases. After the removal of duplicate records, a total of 122 records remained, out of 114 were excluded after title and abstract screening for failure to meet the inclusion criteria. Full-text evaluation was conducted on the remaining 8 articles, of which 5 records satisfied the eligibility criteria and were included in the systematic review.

Three of the included 5 studies were cross-sectional in nature and remaining 2 studies were cohort studies. The Cohen’s kappa coefficient was 0.919 for the inclusion of eligible articles and 1.0 for the data extraction process.

### Risk of bias assessment

The risk of bias assessment scales can be seen in Tables 1 and 2. None of the studies were deemed to be of poor quality. Two studies were classified as fair quality [37, 45] a further 2 of them were classified as good quality [38, 46]. Only one included study was considered very good quality [14] (Table 3).

**Table 1:** Newcastle—Ottawa Quality Assessment Scale (an adapted version for cross-sectional studies) (Herzog et al. [44])

Representativeness of the sample:	Sample size:	Non-respondents:	Ascertainment of the exposure (risk factor):	The subjects in different outcome groups are comparable, based on the study design or analysis. Confounding factors are controlled	Assessment of the outcome:	Statistical test:
a) Truly representative of the average in the target population. * (all subjects or random sampling) b) Somewhat representative of the average in the target population. * (non-random sampling) c) Selected group of users d) No description of the sampling strategy	a) Justified and satisfactory. * b) Not justified	a) Comparability between respondents and non-respondents characteristics is established, and the response rate is satisfactory. * b) The response rate is unsatisfactory, or the comparability between respondents and non-respondents is unsatisfactory c) No description of the response rate or the characteristics of the responders and the non-responders	a) Validated measurement tool. ** b) Non-validated measurement tool, but the tool is available or described.* c) No description of the measurement tool	a) The study controls for the most important factor (RA). * b) The study control for any additional factor (age). *	a) Independent blind assessment. ** b) Record linkage. * c) Self report. * d) No description	a) The statistical test used to analyze the data is clearly described and appropriate, and the measurement of the association is presented, including confidence intervals and the probability level (p value). * b) The statistical test is not appropriate, not described or incomplete

*RA* rheumatoid arthritis. Very good quality: 9–10 points (*), good quality: 7–8 points (*), fair quality: 5–6 points (*), poor quality: 0 to 4 points (*)

**Table 2:** Newcastle–Ottawa Scale for the assessment of the cohort studies

Domain 1				Domain 2	Domain 3		
Representativeness of the exposed cohort	Selection of the non exposed cohort	Ascertainment of exposure	Demonstration that outcome of interest was not present at start of study	Comparability of cohorts on the basis of the design or analysis	Assessment of outcome	Was follow-up long enough for outcomes to occur	Adequacy of follow up of cohorts
a) Truly representative of the average exposed group in the community * b) Somewhat representative of the average exposed in the community * c) Selected group of users e.g. nurses, volunteers d) No description of the derivation of the cohort	a) Drawn from the same community as the exposed cohort * b) Drawn from a different source c) No description of the derivation of the non exposed cohort	a) Secure record (e.g. surgical records) * b) Structured interview * c) Written self reportd) no description	a) Yes * b) No	a) Study controls for the systemic disease other than RA or presence of PP (select the most important factor)* b) Study controls for any additional factor * (age)	a) Independent blind assessment * b) Record linkage * c) Self report d) No description	a) Yes (select an adequate follow up period for outcome of interest) * b) No	a) Complete follow-up—all subjects accounted for * b) Subjects lost to follow up unlikely to introduce bias—small number lost ≥ 80% (select an adequate %) follow up, or description provided of those lost) * c) Follow-up rate < 80% (select an adequate %) and no description of those lost d) No statement

*RA: Rheumatoid Arthritis, PP: Pulpal-periapical Pathology*

Good quality: 3 or 4 stars in selection domain AND 1 or 2 stars in comparability domain AND 2 or 3 stars in outcome/exposure domain

Fair quality: 2 stars in selection domain AND 1 or 2 stars in comparability domain AND 2 or 3 stars in outcome/exposure domain

Poor quality: 0 or 1 star in selection domain OR 0 stars in comparability domain OR 0 or 1 stars in outcome/exposure domain

**Table 3:** Characteristics of the included studies

Study ID	Title	Source (journal, year; volume: pages)	Type of study design	Population	Diagnostic criteria for PP	Association between RA and PP	Results	Quality rating
Jalali et al. 2017 [38]	Prevalence of Periapical Rarefying Osteitis in Patients with Rheumatoid Arthritis	Journal of Endodontics (2017) 43(7) 1093–1096	Cross-sectional	*N* = 262 patientsC = 131 RA = 131	Periapical radiograph Panoramic radiograph PAI score	No	The prevalence of periapical rarefying osteitis is not significantly different in patients with RA compared with control subjects	Good quality
Karatas et al. 2020[12, 14]	Association between Rheumatoid Arthritis and Apical Periodontitis: A Cross-sectional Study	European Endodontic Journal (2020) 22;5(2):155–158	Cross-sectional	*N* = 96 patients C = 48 RA = 48	Periapical radiograph PAI score Clinical examination	Yes	RA is significantly associated with an increased prevalence of AP	Very good quality
Rotstein et al. 2021	Prevalence of periapical abscesses in patients with rheumatoid arthritis. A cross sectional study	American journal of dentistry (2021) 34(4), 211–214	Cross-sectional	*N* = 1,679,976 patients C = 1,669,904 RA = 10,072	Clinical examination Imaging data	Yes	The prevalence of periapical abscesses is significantly higher in patients with RA	Fair quality
Ideo et al. 2022 [37]	Prevalence of Apical Periodontitis in Patients with Autoimmune Diseases under Immunomodulators: A Retrospective Cohort Study	Journal of Endodontics (2022) Jun;48(6):722–729	Cohort study	*N* = 198 patients C = 99 RA = 24 AD = 75	Periapical radiograph PAI score Clinical examination	Yes	Patients with Autoimmune Diseases had a higher prevalence of AP	Fair quality
Heikkila et al. 2022 [46]	Oral health associated with incident diabetes but not other chronic diseases: A register-based cohort study	Frontiers Oral Health (2022); 3: 1–12	Cohort study	*N* = 47681 patients C = 47569 RA = 112	Intraoral radiographs Panoramic radiographs Clinical examination	No	Oral health indices were related to diabetes but not to other chronic conditions (RA)	Good quality

*C* control, *RA* rheumatoid arthritis, *PP* pulpal-periapical pathology, *AP* apical periodontitis, *AD* autoimmune disease

### Population

A total of 1,728,713 patients were examined in the 5 included studies [14, 37, 38, 45, 46]. Of these, 1,717,751 were control patients and the other 10,387 were the RA patients. All the studies reported the mean age and male/female ratio of the patients except 1 study which did not report the mean age of the included patients [45].

### Association between RA and PPP

In one study the number of patients with both RA and AP was unclear, thus corresponding authors were contacted [37]. Following correspondence, it was reported that in the study conducted by Ideo et al. [37] there were 18 patients with AP in a total of 24 RA patients. Table 3 shows the characteristics and the results of the included studies. Out of the 5 studies, 3 reported significant association between RA and PPP [14, 37, 45]. Two of these 3 studies reported association between the RA and AP, the third demonstrated an association between the RA and periapical abscess. The remaining studies reported statistically insignificant association between RA and PPP [38, 46]. Overall, the studies reported a prevalence of AP between 1.53 and 75% in the RA group.

## Discussion

It has been reported that the cytokine profiles including interleukin (IL)-1, IL-6, IL-12, IL-17, tumour necrosis factor α, RANK, and RANK ligand in the pathology of RA and AP are similar [32–36]. Additionally, detection of both the IgG rheumatoid factor and the free rheumatoid factor in periapical lesions of patients with RA might demonstrate certain features of rheumatoid-like inflammation occurs in periapical lesions [47, 48]. Moreover, it is accepted that progression of an autoimmune disease is significantly affected by the systemic inflammatory condition [49]. AP has been associated with increased systemic inflammation [9], which may also support the biological plausibility for such an association. For these reasons, the present systematic review focused on any associations between RA and PPP.

Previously, a systematic review conducted by Guerrero-Gironés et al. [39] evaluated the association between autoimmune diseases and AP, and suggested that there could be an association between autoimmune diseases and AP. They included studies that evaluated the possible association between AP and autoimmune diseases including inflammatory bowel disease, diabetes mellitus, and rheumatoid arthritis. This assertion supported the need for further work in the field to determine where such associations may exist and to explore the strength of associations.

There are > 80 types of autoimmune diseases [50]. Autoimmune diseases are characterised by a breakdown in immune regulation that makes the immune system “auto-aggressive” and fails to distinguish self from non-self [50, 51]. However, different autoimmune diseases have different pathogeneses and nuanced complexities. Therefore, the theoretical and biological plausibility for each specific condition needs to be considered regarding potential associations with AP. To the authors’ knowledge this is the first systematic review aimed to correlate the possible association between RA and PPP.

The studies undertaken to assess whether PPP was linked to RA had contrasting outcomes. Jalali et al. [38] conducted a cross-sectional study and found that the association between RA and periapical rarefying osteitis was insignificant. This was corroborated by another study [46] that reported an insignificant association between RA and AP. The cohort consisted of 47,681 participants and the quality of the study was good according to the current risk of bias assessment. In contrast, a cross-sectional study here the risk of bias assessment showed it was a good quality study [14] reported that there was a significant association between RA and AP. Similarly, a significant association between RA and AP was identified in further research [37]. Additionally, Rotstein and Katz [45] conducted a cross-sectional study with more population consisting of 1,679,976 participants which reported that the prevalence of periapical abscesses was significantly higher in patients with RA than in the control. Differences in the study type and sample size may be the cause of disagreement among the studies. Additionally, these disagreement among the included studies may be attributed to methodological differences in particular the diagnosis of AP. Accurate diagnosis of AP requires both clinical and radiographic examinations [5]. However, in the study conducted by Jalali et al. [38] the diagnosis of AP was made based solely on radiographic examination. It is accepted that radiographic examination alone may not be sufficient for accurate diagnosis of AP [52] as periapical lesions confined to cancellous bone cannot always be accurately detected radiographically. Cortical bone involvement facilitates more accurate diagnosis [53, 54]. Participant characteristics are a further potential source of dissonance amongst the included studies. In the study conducted by Rotstein and Katz [45], the control group consisted of hospital patient population. This will likely include participants with systemic diseases including those that have also been associated with AP, and thus result in a degree of confounding.

The studies included in the present systematic review, were evaluating the association between RA with endodontic conditions. Different endodontic diseases display different pathological, radiographic and clinical characteristics [55]. Given that there was variation in the particular endodontic condition considered within the studies, this may further account for some of the variation in results. Jalali et al. [38] investigated the possible association between RA and periapical rarefying osteitis, Rotstein and Katz [45] investigated the association between RA and periapical abscess. The remaining 3 included studies [14, 37, 46] evaluated the possible association between RA and AP. Since the present systematic review aimed to investigate possible association between RA and endodontic disease, the main inclusion criterion of the present work was the studies evaluating PPP. Thanks to this inclusion criterion, endodontic disease with different clinical and radiographic characteristics were included in the present systematic review.

It should be taken into account that the number of studies included in the present systematic review are limited. Currently, prospective clinical trials on this topic do not exist and the majority of the included studies are observational in which validity issues, confounding, and bias are common [56]. Additionally, cohort studies do not have the highest evidence level to provide casual association [57]. Therefore, prospective parallel-group studies of RA patients and healthy patients, investigating prospectively the incidence of new PPP would provide more evidence for potential associations between RA and PPP.

## Conclusion

On the basis of the existing evidence an association between RA and PPP is possible. However, it is hard to draw firm conclusions based on the number, quality, and heterogeneity of the studies included to the present systematic review. Thus, further high-quality primary research evaluating the possible association between RA and PPP are needed.

## Data Availability

The data that support the findings of this study are available on request from the corresponding author.
